# Cassava root crown phenotyping using three-dimension (3D) multi-view stereo reconstruction

**DOI:** 10.1038/s41598-022-14325-4

**Published:** 2022-06-15

**Authors:** Pongsakorn Sunvittayakul, Piya Kittipadakul, Passorn Wonnapinij, Pornchanan Chanchay, Pitchaporn Wannitikul, Sukhita Sathitnaitham, Phongnapha Phanthanong, Kanokphu Changwitchukarn, Anongpat Suttangkakul, Hernan Ceballos, Supachai Vuttipongchaikij

**Affiliations:** 1 Department of Genetics, Faculty of Science, Kasetsart University, 50 Ngam Wong Wan Road, Chatuchak, Bangkok, 10900 Thailand; 2 Department of Agronomy, Faculty of Agriculture, Kasetsart University, 50 Ngam Wong Wan Road, Chatuchak, Bangkok, 10900 Thailand; 3 Center for Advanced Studies of Agriculture and Food (CASAF), Kasetsart University, 50 Ngam Wong Wan Road, Chatuchak, Bangkok, 10900 Thailand; 4 Center of Advanced Studies for Tropical Natural Resources, Kasetsart University, 50 Ngam Wong Wan Road, Chatuchak, Bangkok, 10900 Thailand; 5 Omics Center for Agriculture, Bioresources, Food and Health, Kasetsart University (OmiKU), Bangkok, Thailand; 6 The Alliance of Bioversity International and the International Center for Tropical Agriculture (CIAT), Cali, Colombia

**Keywords:** Biological techniques, Plant sciences

## Abstract

Phenotypic analysis of cassava root crowns (CRCs) so far has been limited to visual inspection and very few measurements due to its laborious process in the field. Here, we developed a platform for acquiring 3D CRC models using close-range photogrammetry for phenotypic analysis. The state of the art is a low cost and easy to set up 3D acquisition requiring only a background sheet, a reference object and a camera, compatible with field experiments in remote areas. We tested different software with CRC samples, and Agisoft and Blender were the most suitable software for generating high-quality 3D models and data analysis, respectively. We optimized the workflow by testing different numbers of images for 3D reconstruction and found that a minimum of 25 images per CRC can provide high quality 3D models. Up to ten traits, including 3D crown volumes, 3D crown surface, root density, surface-to-volume ratio, root numbers, root angle, crown diameter, cylinder soil volume, CRC compactness and root length can be extracted providing novel parameters for studying cassava storage roots. We applied this platform to partial-inbred cassava populations and demonstrated that our platform provides reliable 3D CRC modelling for phenotypic analysis, analysis of genetic variances and supporting breeding selection.

## Introduction

Cassava (*Manihot esculenta* crantz), a major root crop in the tropical and subtropical regions, is an important supply for food, animal feed and starch-related industries worldwide^1,2^. The cassava root system consists of adventitious roots developed from the nodes and the base of stem cuttings and lateral roots during the early establishment of the crop^3^. Starting 2–3 months after planting (MAP), some of the fibrous roots form storage roots in concomitant with drastic decreases of adventitious and lateral roots during the development towards crop maturity^4^. Indeed, cassava productivity has been improved through extended breeding programs in the past several decades, by selecting high-performance varieties with large root crowns and high starch content yet resistant to pests, diseases and environmental challenges^5,6^. However, cassava breeding practices in most countries are still carried out through visual selections and evaluation on-site^7,8^. These processes are labor-intensive with a limited operating time as cassava samples deteriorate within a few days after separation from the main crown. Data obtained on the field are usually restricted to a few characteristics such as approximate root sizes, number of roots and weight, dismissing detailed cassava root crown (CRC) parameters for an in-depth phenotypic analysis. In the era of precision breeding, tools that allow detailed and systematic data collection in the field are critically needed^9,10^.

Visual-based detections are a powerful tool for the acquisition of plant phenotypes. In recent years, two-dimension (2D) image-based object detection and segmentation systems have been developed for recording plant phenotypes^11^ as well as those of cassava roots^12,13^. Although they can be easily used on-field, the 2D data usually suffer from occultation and ambiguity caused by perspective projection^14^, limiting the data accuracy and numbers of analysis parameters. For example, the CRC has to be separated into individual roots for photography, resulting in the crown shape and size loss. These parameters, in turn, are critical for the emerging technologies aiming at the mechanical harvest of the crop. Three-dimension (3D) visualization and reconstruction tools are emerging platforms for virtual morphological characterizations of different plant species^15^. However, CRCs have a distinct and complex 3D structure from other crop roots^16^. Therefore, it is challenging to apply these available platforms directly to the CRC, let alone their application in the field.

Different imaging tools for reconstructing 3D models from plant samples have been developed, including structural light spectroscopy, thermal imaging and photogrammetry^17^. Alternatively, non-invasive data acquisitions of 3D root structures could be achieved from plants grown in containers through X-ray tomography, MRI and X-ray CT scanning^18–20^. However, most of these tools require a controlled environment such as a scanning studio, a long acquisition time per sample or high-cost equipment set up, all of which is incompatible with an on-field experiment. Among these tools, 3D-photogrammetry has been developed to align multiple 2D images and convert them into a 3D point cloud model^21^. This tool is particularly attractive for acquisitioning the 3D CRCs as photo shooting is adaptable to different field conditions.

Field assessment is essential for studying cassava traits involved in root production and understanding environmental factors at the molecular levels^22,23^. However, there are specific requirements for cassava field-trials. CRCs must be excavated for visual inspection and data collection right on the day as the roots start losing water content on the open field. Furthermore, the field-trials are usually performed in remote rural areas, without on-site power supply, requiring either transporting equipment to the field or the intact root crowns to the lab, which is labor-intensive and expensive. A platform for collecting the 3D information for CRCs has to be a simple and fast process, with minimal hardware setup for convenient maneuvers and without requirements for direct power lines. Based on the nature of cassava field experiments, we propose using 3D photogrammetry with 2D images as a suitable field-adaptable phenotyping tool for CRCs.

Here, we developed an adaptable and economical platform for acquiring 3D images of CRCs for a detailed evaluation of root phenotypes for precision breeding, agronomy and crop physiology. 3D analysis was verified and optimized for accurate measurements of CRCs. We applied our platform for phenotypic analysis of breeding populations in the field and showed that genetic segregations could be assessed through the analysis of 3D traits.

## Methods

### Plant materials, trial conditions and data collection

CRCs used for 3D reconstructions in this work were obtained from two separated experiments: 19 randomly selected CRCs from a selection trial for method optimization and partial-inbred populations of KU50 for phenotyping study. KU50 is a released Thai cultivar^5,24^. S_1_ and S_2_ partial-inbred lines from KU50 were generated by controlled self-pollination. Stem cuttings (20-cm long) were planted in a single row trial (SRT) with 1.5 × 1.5 m spacing (within and between rows) using an augmented randomized complete block design (augmented RCBD) with nine blocks and non-replicated samples. Each block contains five commercial varieties used as checks. Each row contained eight cutting stems, and the middle six plants were used for data collection. Border rows were applied. The experiment was performed in Photharam District, Ratchaburi, Thailand (location coordinate: 13.653699 and 99.821265) from April 2019 to March 2020 without irrigation supply. The soil type is fine-sandy loam. Fertilizers were added at 4 MAP. Environment conditions including rainfall and temperature are present in Supplementary Table S1. Cassava was harvested at 11 MAP. All methods were performed in accordance with the relevant guidelines and regulations.

CRCs were manually excavated using a leverage tool with an attached grip; the stem base was locked within the grip before lifting the whole crown from underground. The CRCs were carefully pooled out of the ground to prevent root breakages as much as possible. The crowns were briefly cleaned using a brush, weighed and photographed with the setup described below. Harvest index (HI) was determined on the field as a root weight ratio to the whole plant weight. Dry matter content (DMC) is expressed as a percentage of dry to fresh root mass. In this case, 300 g of thin fresh-root slices (~ 5 mm using a food slicer) were obtained from the middle section of three selected cassava roots per crown, placed in a paper bag, dried in an incubator oven at 70 °C for 7 days and weighed again using a two-digit balance. The remaining percentage was regarded as moisture content. Starch content (% w/w) was analyzed from dry root samples in triplicates using the total starch assay kit (Megazyme, Bray, Ireland).

### Hardware, software and photography

DSLR cameras, including Nikon D5300, Cannon EOS750D and EOS450D were used for photography. Agisoft Metashape 1.6.5 standard edition^25^ with an educational license was used for photogrammetry, and Blender 2.90.1^26,27^ with “Mesh: 3D-print toolbox” add-ons was used for 3D analysis. These software packages were installed in a laptop (Intel^®^ Core™ i7-8400 CPU 3.2 GHz, 32 GB RAM and NVIDIA GeForce GTX 1070 GPU), which was used throughout this work. The camera’s parameters were adjusted as follows; the highest F-stop with a small aperture, high depth of field, ISO speed < 400 to prevent image noise, auto white balance, medium image size (< 5 MB) and no flash mode. For photo shooting, the entire CRC was placed on its side exposing the root structure on a green background (120 × 120 cm) with a cardboard box (W × L × H: 12.5 × 12.5 × 34 cm) as a 3D reference object. Photographs were taken by stepping around the root crown to obtain 25–40 images per object. Oculus Quest 2 was used as a virtual reality tool in Medium by Adobe (version 2.4.6.336).

### 3D reconstruction of CRC models

Images were imported to Agisoft for photo alignments (~ 15 min per CRC). A working region covering the CRC and reference object can be selected to build a dense cloud (~ 25 min) and subsequently build a mesh (~ 15 min). The mesh was then subjected to manual noise clearing and background removal to obtain a clean 3D model before exporting as a .obj file (see Supplementary Information 2 as .obj and Supplementary Video 1as a sample). This 3D reconstruction process is demonstrated in Supplementary Video 2.

### Analysis of 3D models and data validations

A .obj file was imported to Blender 2.90.1. Holes were closed by choosing “Add modifier” then “Remesh” with the “Smooth” and “Ortree depth 8” options. The models were then rescaled based on the reference object using “Scene properties”. Before calculating the volume, 3D models were separated in half to decrease the bias volume from the photogrammetry process. The reference object was deleted before computing the volume and area via Mesh 3D-print toolbox add-on. 3D traits were collected using measurement and analysis tools. For the dataset used for correlation analysis, root angle and root length were averaged from every root contained within each CRC. For root angle in partial-inbred populations, root angle was averaged from three lowest angles within each CRC. For data validation, whole root crowns and individual storage roots were collected from fields for photo shooting and direct measurements. Root lengths were measured using a ruler tape. Roots or crowns samples were entirely immersed in water in an overflowing cylinder, and water replacement volumes were regarded as root or crown volumes.

### Data analysis

Analysis of different phenotypic values obtained from S_1_ and S_2_ generations was performed using an augmented RCBD package (Aravind et al.^28^) in R. The normal distribution of 3D CRC was tested using a normal quantile–quantile plot in R. Correlation analysis of different trait parameters was analyzed using the PerformanceAnalytics package^28^ using the correlation matrix chart function. Principal component analysis (PCA) was computed using FactoMineR and factoextra R package. Broad sense heritabilities were calculated following the method used by Falk et al*.*^29^.

## Results

### 3D reconstructions of CRCs

**Figure 1:**
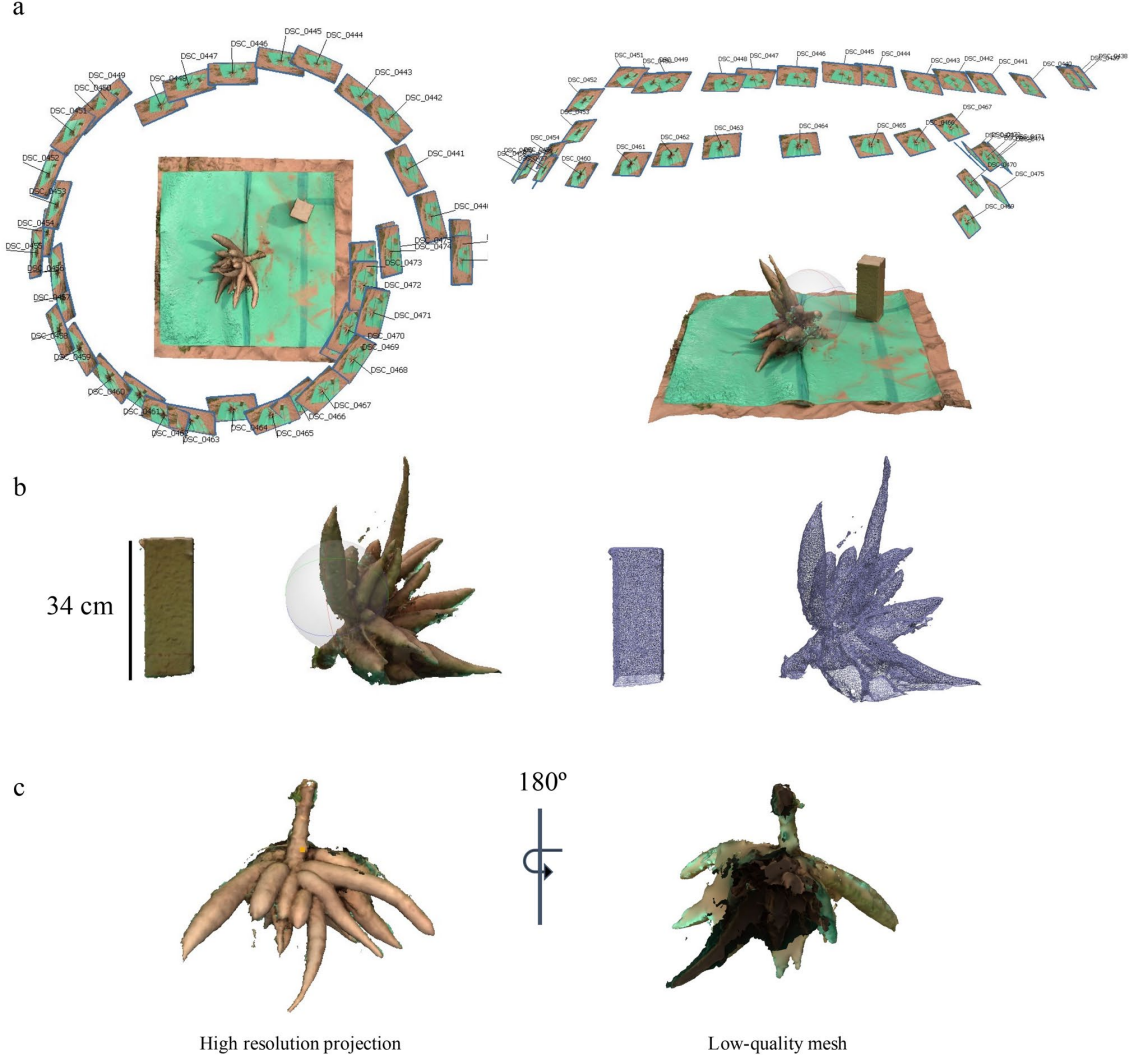
A 3D reconstruction of a CRC from 2D images using Agisoft Metashape Standard 1.6.5 (http://www.agisoft.com/downloads/installer/). (**a**) A representative image for 3D CRC reconstruction through 360° photo alignment, dense cloud and 3D mesh generation with different camera projections. The root object was placed on a green background with a reference object using a card box (W × L × H: 12.5 × 12.5 × 34 cm). (**b**) An exported .Obj model file with background removed and noise clearing for Blender. (**c**) Projections of 3D root crown at high resolution and low-quality mesh from the blind area.

**Figure 2:**
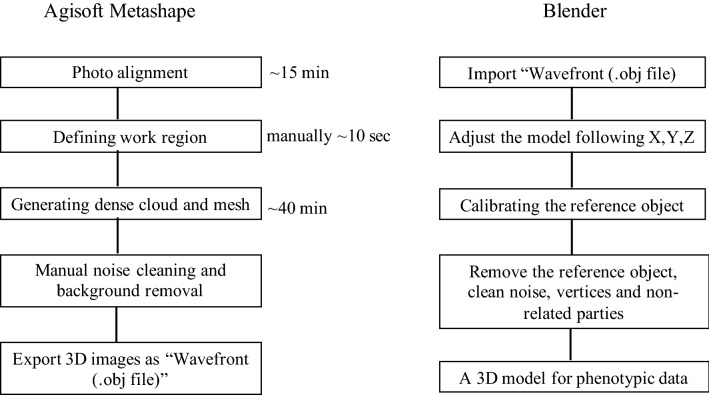
Workflows for 3D CRC reconstruction in Agisoft and 3D model analysis in Blender. An approximate processing time for each step is indicated.

To generate 3D CRC models, we acquired ~ 40 photographs from each freshly-harvested CRC in the field using a DSLR camera. CRCs were laid on their side to expose the root structure on a green plastic sheet as background and a cardboard box as a size reference for the photo shooting (Supplementary Fig. S1). Initially, we tested five well-recognized 3D reconstruction software packages, either a free license or free trial, to generate root crown models. Using the same datasets, Agisoft provided the best quality dense cloud (~ 6000 points) and 3D mesh with a realistic look, while other packages failed or provided low-quality 3D meshes (Supplementary Fig. S2). A photo alignment and 3D model generated by Agisoft are presented in Fig. 1. At this stage, the 3D image for a CRC can be observed through 3D rotation within the software environment. It is important to note that some areas of the 3D models are resulted from low-quality meshes (Fig. 1c), which were generated from the blind areas of root crowns placed on the background surface. The 3D image as a wavefront file (.obj file format) can be used for various applications in other 3D software packages. The workflow is summarized in Fig. 2.

**Figure 3:**
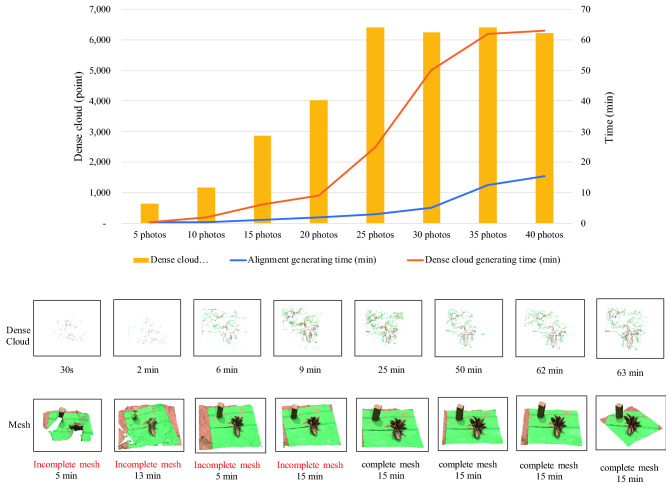
An optimization of the number of images for 3D reconstruction using Agisoft Metashape Standard 1.6.5 (http://www.agisoft.com/downloads/installer/). The numbers of images (5, 10, 15, 20, 25, 30, 35 and 40) were taken from the same root crown and processed through image alignment, dense cloud generating and mesh generating. Time used each processing step, dense cloud points and mesh qualities are presented.

We verified a minimum number of 2D images required for generating a 3D mesh by using 5 to 40 images as input datasets and dense cloud point, processing time and finished mesh as testing parameters. We found that at least 25 images are required for generating a high-quality 3D root crown mesh with a maximum cloud point and ~ 45 min for processing times for photo alignment, dense cloud and mesh generations (Fig. 3). Moreover, Agisoft has a built-in tool for batch processing of multiple datasets, allowing a high throughput 3D reconstruction of CRCs. We were able to process 100 cassava datasets yielding 100 individual 3D root crowns within three days following the described protocol. These results demonstrate that 3D CRC models can be efficiently reconstructed by Agisoft using 2D images acquired on the field. The built 3D meshes could be observed, analyzed and manipulated in the 3D environment. In addition, we also tested the 3D reconstruction using photos taken by a mobile phone and found that the mesh quality and cloud points were comparable to those obtained from DSLR cameras.

### Analysis of 3D CRCs and validations

**Figure 4:**
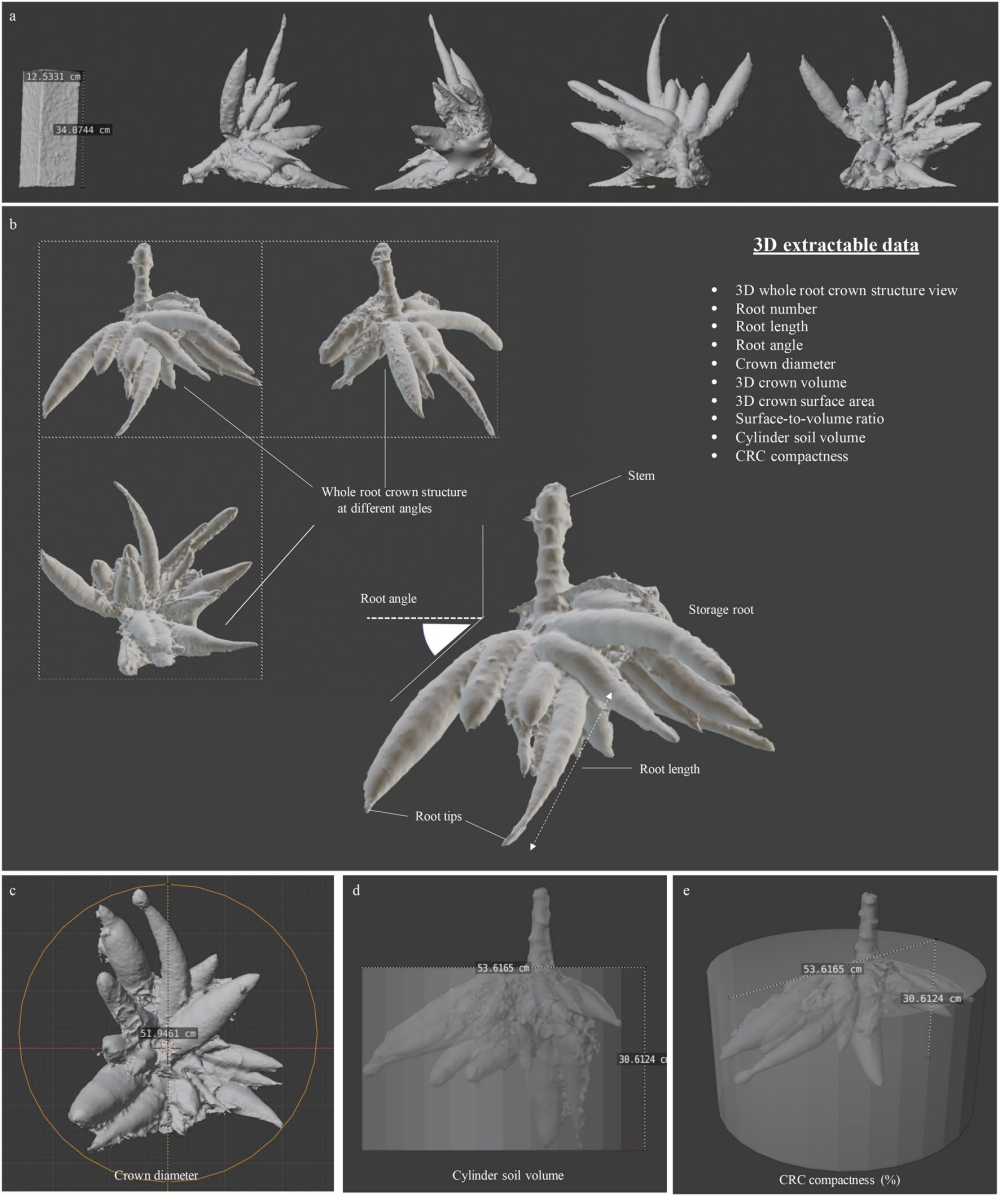
Representative of 3D root crowns in Blender environment for data analysis (Blender 2.90.1: http://www.blender.org). (**a**) Directional arrangements of a 3D root crown model and a scale calibration using the refence object. (**b**) Extractable 3D traits obtained from the model. (**c**) A measurement of root crown diameter. (**d**) a measurement of cylinder soil volume. (**e**) CRC compactness represents a percentage of a crown volume within a cylinder soil volume.

**Figure 5:**
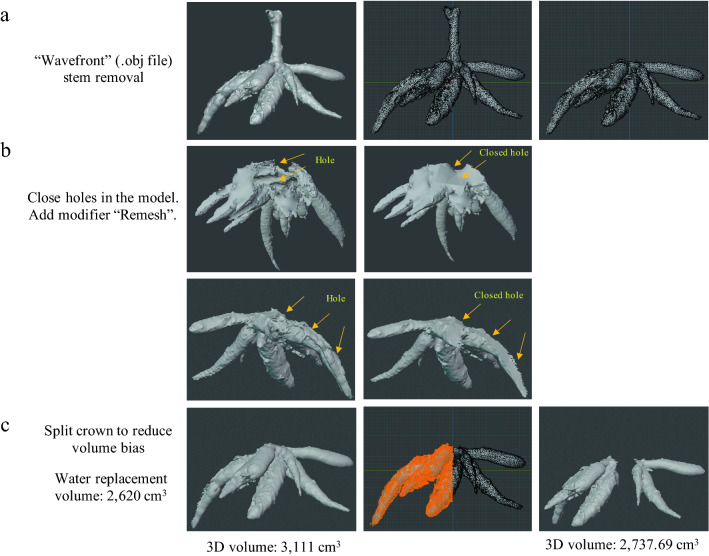
3D object manipulations for root crown measurements in Blender environment (Blender 2.90.1: http://www.blender.org). (**a**) The stem section is manually removed from the root crown. (**b**) Holes in the object surface are closed using a “Remesh” tool in Blender. Two representatives of hole closing are presented. (**c**) A 3D crown is manually split in half to reduce volume bias. Crown volumes obtained from water replacement, intact crown and split crown are indicated.

We tested five different 3D software packages and found that Blender has easy-to-use tools for manipulating the CRC and calculating the object size and volume than other tested software (Supplementary Fig. S3). The workflow for processing the 3D root crown in Blender is summarized in Fig. 2 (see Supplementary Video 3 for a movie clip). Figure 4 summarizes CRC phenotypes instantly obtained from a 3D model in Blender, including the whole CRC structure, root numbers, root angle, root crown diameter, cylinder soil volume and root length (Fig. 4b,c). The resolution of the 3D root crowns is at millimeter scale. However, measurements of 3D crown volumes and crown surface areas require object manipulations before computational quantification (Fig. 5). The stem section of CRCs must be removed to avoid unwanted data, and the root surfaces must be sealed off using the “Re-mesh” tool. At this stage, the 3D crown volume and crown surface area can be calculated by the software using the “Analysis” tool.

Subsequently, the primary data obtained above can be used for calculating additional parameters. Firstly, root density (root weight/3D volume) helps to indicate the levels of starch and potentially other compounds accumulated in the roots. Second, a surface-to-volume ratio represents root shapes: a lower ratio indicates shorter or thicker roots, whereas a higher ratio indicates longer or thinner roots. Lastly, CRC compactness, calculated as a percentage of 3D crown volume per its fitted cylinder soil volume, allows assessments of the crown shape and effectiveness in soil use.

**Figure 6:**
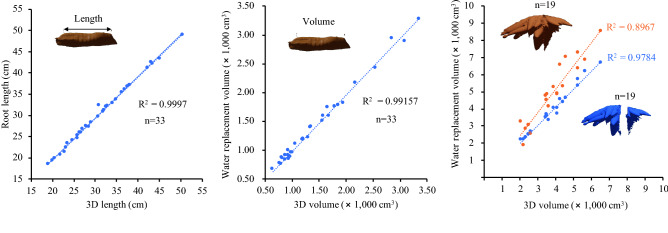
Validation of measurements for lengths and volumes of cassava roots and root crowns. Scatter plots show data for ground truth measurements and 3D calculations for root lengths, root volumes and root crown volumes, respectively. For the root crown volume, intact and split crowns are compared.

We collected a number of cassava samples from a field trial and ran through the platform to validate the collected 3D data against the ground truth data. CRC architecture and size varied considerably. While the 3D measurements for root lengths and volumes were accurate (Fig. 6), the measurements for 3D crown volumes were obtained with relatively low accuracy (R^2^ = 0.89668). A number of factors affecting the accuracy include the limited data at the blind area and spaces within the crown, amalgamated in the computation. Refining the 3D models and removing noises did not help. Surprisingly, when the 3D models were digitally split in half (Fig. 5c), accurate measurements of the 3D crown volumes were obtained (R^2^ > 0.97 with an error rate within 10%) (Fig. 6c, Supplementary Table S2). We have included a video file for this process (Supplementary Video 4). However, we did not validate every measurement presented here. This is because the validations for the length and volume can be applied to other length- and volume-based parameters. Furthermore, it is extremely difficult to obtain ground truth data for root angle and crown surface area, and, thus, we rely on the Blender software algorithm for the accuracy of measurements. Because there is currently no software available for extracting these phenotypic data from the 3D model, these data were manually recorded. Moreover, 3D CRCs can be digitally dissected into individual roots, which allow detailed measurements of the length and diameter of each root (Supplementary Fig. S4). Because there is currently no software available for extracting these phenotypic data from the 3D model, these data were manually recorded. Lastly, we show that a virtual reality (VR) tool can be help to observe the 3D CRCs (Supplementary Video 5). These results demonstrate that detailed phenotypic analysis of CRCs can be obtained through our platform.

### Correlation analysis of phenotypic data from 3D root crowns

**Figure 7:**
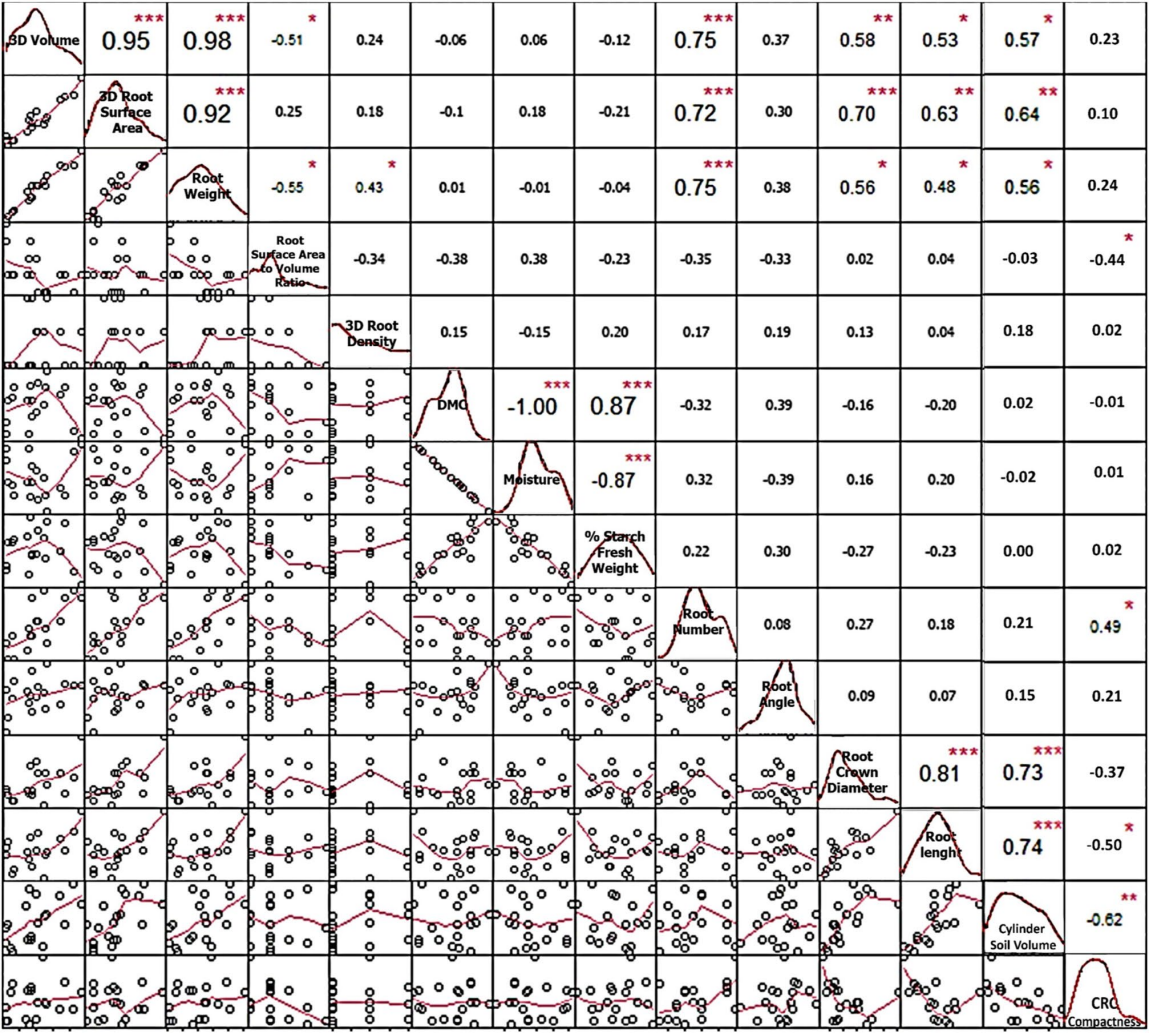
Correlation plots among 3D crown volumes (cm^3^), 3D crown surface areas (cm^2^), root weights (kg), surface-to-volume ratios, root density, DMC (%), moisture (%), starch content (% fresh weight), root number, root angle, root crown diameter (cm), average root length (cm) cylinder soil volume and CRC compactness obtained from 19 CRCs of seven cassava genotypes (df = 17). *, **, *** represent significant at P < 0.05, 0.01 and 0.001, respectively.

Correlation analysis of the phenotypic data (Supplementary Table S2) showed that 3D crown volumes, 3D crown surface areas and root weights correlate tightly to one another (P < 0.001) and significantly correlate with the root numbers (P < 0.001), crown diameters (P < 0.05), average root lengths (P < 0.05) and cylinder soil volume (P < 0.05) (Fig. 7). The data indicate that measurements of 3D volumes and 3D crown surface areas computed from 3D models are strongly supported by the root weight data, and all the above parameters correlate to the size of CRCs. The CRC compactness correlates with the root number (P < 0.05) and indeed negatively correlates with the average root length and the surface-to-volume ratio (P < 0.05). No correlation was observed for root angle. It is apparent that parameters regarding the volume and sizes do not correlate to those for the root content, including root density, DMC, moisture and starch content. This observation supports the notion that traits related to the crown and root sizes and their contents are inherited independently. Initially, we tried to see if the density could be used to determine the starch content, commonly measured by a specific gravity method using the fresh roots^30^. However, in our case, the density did not correlate to starch content and, thus, cannot be used to determine the starch content.

### 3D root crown analysis of partial-inbred populations

We applied our platform to assess partial-inbred populations. S_1_ lines were pre-selected from 248 botanical seeds, later 92 seedlings and finally 29 vigorously grown plants. Twelve S_2_ lines were obtained solely from a single KU50-S_1_ line. ANOVA showed significant differences among genotypes for the 3D crown volume, 3D crown surface area, surface-to-volume ratio, root weight, root density, root number and cylinder soil volume (P < 0.05) (Supplementary Tables S3, S4).

**Figure 8:**
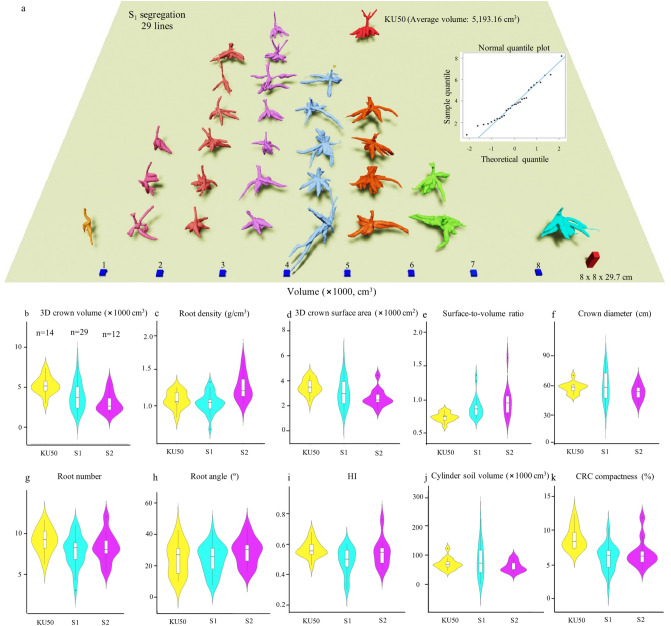
Phenotypic analysis using the 3D platform for cassava inbreeding populations. (**a**) 3D models representing 29 S_1_ lines fitted into a normal distribution based on average 3D crown volumes, as supported by a normal quantile plot. KU50 as the S_0_ progenitor is presented. Volume scales are indicated with blue boxes. A reference object is included. (**b–k**) Box and whisker plots and violin plots overlaid present phenotypic segregations of S_1_ and S_2_ inbreeding populations compared to KU50 for nine 3D traits and harvest index.

**Figure 9:**
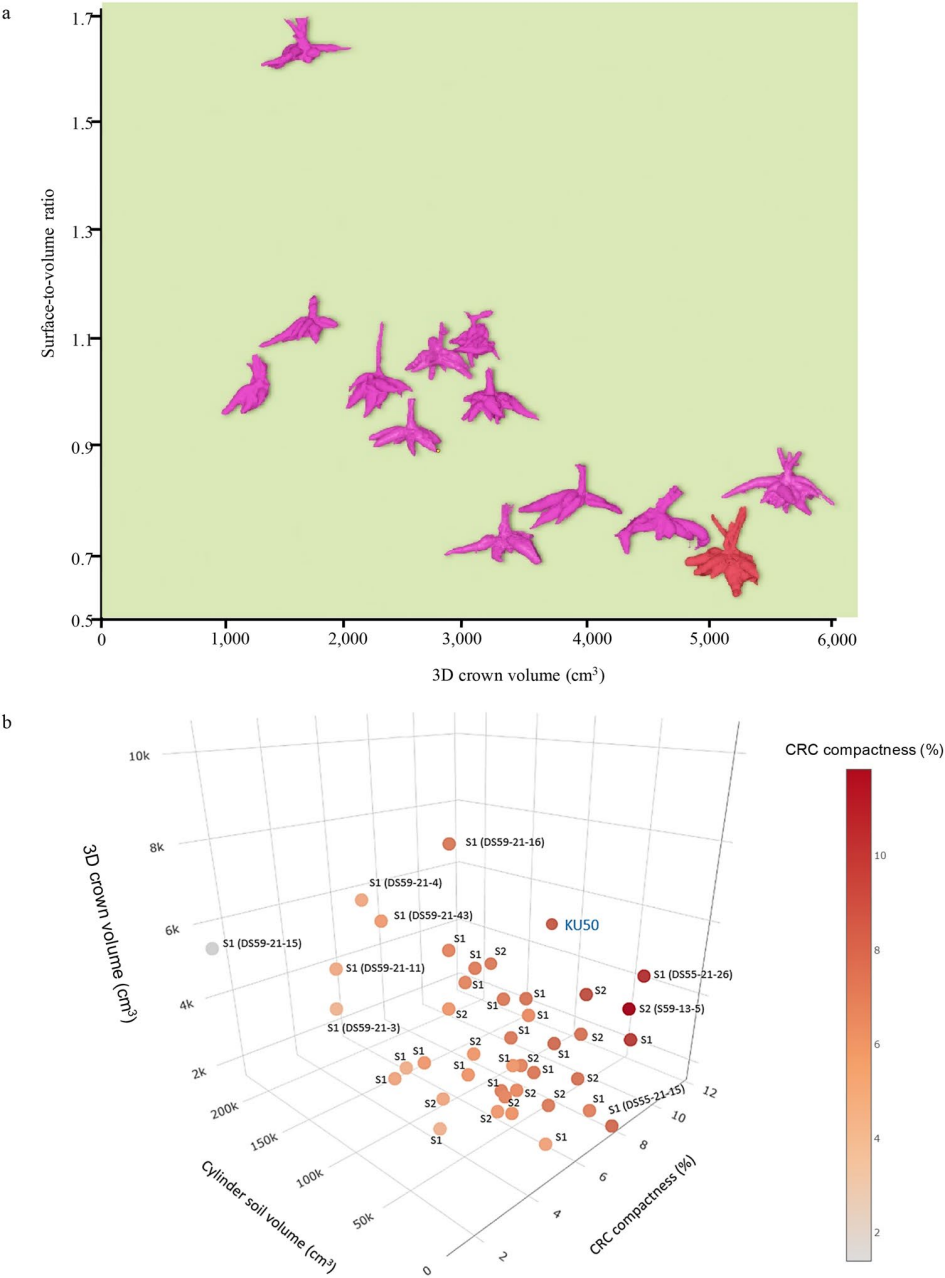
(**a**) Segregations of the 12 S_2_ population plotted between for surface-to-volume ratios and 3D crown volumes. Representative 3D CRC models for each S_2_ lines were selected from 3D models with median values. KU50 (S_0_) is presented in red. (**b**) A 3D scatter plot of 3D crown volume, cylinder soil volume and CRC compactness for 29 S_1_, 12 S_2_, and KU50 using adjusted means from augmented RCBD analysis.

Figure 8a shows representative CRC models from 29 S_1_ lines fitted to the normal distribution of the 3D crown volumes, demonstrating the variation of the 3D crown volume trait in S_1_. 3D traits and HI present the segregations of the S_1_ and S_2_ lines to different degrees (Fig. 8b–k). The 3D crown volume, crown surface area, crown diameter and cylinder soil volume of S_1_ lines show wide segregations of these quantitative traits compared to those of the progenitor (KU50), and they were becoming fixed in the S_2_ through the inbreeding process. The root density of S_1_ was similar to that of S_0_ and increased in the S_2_ generation. However, the root number, root angle and HI of the S_1_ lines were somewhat similar or lower than KU50 and S_2_. The surface-to-volume ratio indicates that the S_2_ population segregated towards thinner storage roots (Fig. 9a). For CRC compactness, the S_1_ and S_2_ segregated towards less-compact CRC with some high-compactness individuals. Furthermore, when plotted together the 3D crown volume, cylinder soil volume and CRC compactness, this would allow a selection for CRC volumes, shapes and efficiency of soil use (Fig. 9b).

Principle component analysis (PCA) showed that the first five PCs contributed to more than 93% of the total phenotypic variation (Table 1). The root weight, 3D crown volume, 3D crown surface area, crown diameter, HI, cylinder soil volume, root number and surface-to-volume ratio contributed significantly to variation (P < 0.0001). PCA indicated that these eleven traits are relevant to the phenotypic variations in the populations. Additionally, moderate broad sense heritabilities (> 0.6) were observed for root weight, 3D crown volume, 3D crown surface area, crown diameter and cylinder soil volume (Table 2). This result shows that the 3D traits can be used for assessing genetic segregations in cassava, including those from inbreeding, and 3D crown volume, 3D crown surface area, crown diameter and cylinder soil volume are the most reliable 3D traits to be used primarily for cassava breeding research.

**Table 1:** Principle component analysis and variable contribution of nine 3D traits, root weight and HI.

Variables	PC1	PC2	PC3	PC4	PC5
Root weight	**0.94**	0.15	0.11	0.00	0.07
3D crown volume	**0.92**	** − **0.17	0.23	0.08	** − **0.12
3D crown surface area	**0.92**	** − **0.28	0.15	0.09	0.02
Crown diameter	**0.86**	** − **0.06	** − **0.28	0.07	0.07
HI	**0.70**	**0.60**	** − **0.23	0.11	0.00
Cylinder soil volume	**0.67**	** − 0.48**	** − **0.37	0.26	0.03
Root number	**0.55**	** − **0.32	0.43	** − **0.33	**0.47**
Root density	0.32	**0.77**	** − **0.32	0.09	0.39
CRC compactness	** − **0.04	**0.53**	**0.77**	** − **0.18	** − **0.15
Root angle	** − **0.18	0.05	**0.46**	**0.86**	0.03
Surface-to-volume ratio	** − 0.77**	** − **0.17	0.09	0.13	0.50
Eigenvalue	4.88	1.60	1.29	0.87	0.67
% Variance	48.76	16.02	12.94	8.72	6.74
Cumulative variance (%)	48.76	64.78	77.72	86.44	93.17

Significant variables are in bold with correlation coefficient (r) > 0.450 (P < 0.001).

**Table 2:** Broad sense heritability of nine 3D traits, root weight and HI.

Traits	Broad sense heritability
Root weight	0.61
3D crown volume	0.70
3D crown surface area	0.70
Crown diameter	0.72
HI	0.24
Cylinder soil volume	0.79
Root number	0.37
Root density	0.50
CRC compactness	0.50
Root angle	0.07
Surface-to-volume ratio	0.44

## Discussion

Close-range photogrammetry has been employed for the 3D reconstruction of many plant systems through commercial or custom-designed software^31^. However, 3D modeling of CRCs has not yet been reported, and suitable photogrammetric software for an accurate 3D reconstruction and analysis have to be assessed. Here, Agisoft was selected for the 3D reconstruction based on the quality of 3D CRC models. Agisoft is equipped with four fully automated photogrammetric processes: image alignments using Structure from Motion (SfM) algorithm, dense point cloud generations using a dense image matching (DIM) algorithm, meshing and then texturing. The program is simple to use and does not require computing professionals. The workflow walk-throughs are included, in this report. We typically reconstructed 100 datasets (100 × 25 images) per run with its built-in batch processing. Subsequently, Blender was selected as the most suitable software for modeling and compositional analysis of the 3D CRCs. So far, we have utilized this platform for the streamlined processing of 3D reconstruction and modeling of more than 1000 CRC datasets. Our workflow using the two software packages is similar to a report for modeling weed plants in the field using Agisoft and Meshlab^32,33^. These photogrammetry and modeling software tools should be explored to support 3D applications in other crops.

Although a number of 3D analysis software for object detection, scale calibration, segmentation, estimation and trait classifications through the use of deep learning are being developed for automated extractions of 3D data^34,35^, they are currently inapplicable for the CRC, which requires specific software development. Nevertheless, we employed Blender for manual data extraction from the 3D CRC models, in which the whole 3D root crown structure is an important feature for evaluating root phenotypes. In the areas of automated sensors for agriculture, on-field 3D acquisition and detection are still very challenging^36–39^. Thus, our platform poses as a step towards automated data extraction tools for on-filed cassava phenotyping as our subsequent development. Alternatively, we have acquired VR as a helping tool for observing the 3D CRCs.

Our 3D measurements for lengths and volumes made in Blender have been validated against the ground truths for measurement accuracy. Generally, measurements of individual roots are of higher accuracy than those of the whole CRCs, and this is most likely due to the complexity of the crown structure and the partial loss of information in the blind area. Nonetheless, we found that splitting the crowns in half helps to improve the accuracy (from R^2^ > 0.89 to 0.97). This finding may be related to the resolution enhancement of split large image volumes reported by Blumberg et al.^40^.

Image-based phenotyping of cassava storage roots reported so far is mainly restricted to individual roots, such as root lengths and widths^13^, while the aspects of the whole root crown have not been fully explored. Only root angle and crown diameter parameters, which could be retrieved from 2D photos, have been used to represent CRC data^12^. Furthermore, genome-wide association studies of cassava root traits reported to date only relied on individual root data and none of the whole crown parameters^9,10,41,42^. It is apparent that cassava phenotyping is limited to direct measurements and 2D data, and the whole crowns have never been studied due to limited tools. We demonstrate that 3D CRC models can be acquired in the field through our platform, as shown in the analysis of the partial-inbred populations. In addition to the root angle and crown diameter, our 3D CRC models provide actual measurements of 3D crown volumes, 3D surface areas, root density, cylinder soil volume and CRC compactness, which have never been shown before in other cassava phenotyping platforms. These newly identified parameters as verified through PCA and broad sense heritability would be essential for genome analysis, including GWAS, to identify novel genes involved in the CRC development and cassava root yield. Our platform presents as a package for assessing other root traits typically obtained in the field, including root numbers and root lengths, while analyzing the 3D CRC models. The platform also reduces human errors from direct measurements and data recording in the field.

Typically, root weight and root number, which are simple to measure in the field, are generally employed as the main selective traits in cassava breeding programs^6,8^. Our 3D method provides a tool for recording and measuring the whole intact CRC to individual roots for detailed measurements. Though easy to perform, measurements of crown and root sizes are often neglected due to their laborious process and limited operation time in the field, but now they can be recorded using our platform. Furthermore, parameters associated with volumes, areas and angle that are very difficult to measure can be extracted through the software. Although root weight alone can represent the cassava root yield, there are different traits that contributed the overall root yield, for example root volume, root length and thickness, root number, root crown shape and root density. Dissecting these traits would allow us to study the genetic controls of the different components of cassava root yield, which so far is inconclusive. Analysis of the 3D traits in the partial-inbred populations showed that six traits including 3D crown volume, crown surface area, crown diameter and cylinder soil volume, surface-to-volume ratio and CRC compactness were segregated along inbreeding indicating the genetic variabilities to be potentially used for breeding selection. Furthermore, PCA and broad sense heritability supported that, addition to root weight, 3D crown volume, 3D crown surface area, crown diameter and cylinder soil volume can be used when selecting for root volume, root shape and effective soil use.

Moreover, the 3D models provide detailed features of CRCs for morphological and physiological studies concerning to agronomic aspects, for example, as a tool for assessing environmental effects to the crown shape and size and understanding how the CRC shape could affect cassava yield. For example, the cylinder soil volume and CRC compactness provide a platform for studying cassava crown growth to soil, air and water within an underground space, and soil space required for growing each cassava plant can be assessed. Notably, the 3D model may be used in conjunction with ground penetrating radar (GPR) to support non-destructive predictions of root yield in the field^43^. Various factors from the soil that affect cassava crown formation could be assessed through the change of the CRC shape, size, angle and occupied underground space in 3D as indicated by the CRC compactness and cylinder soil volume. These soil factors may include rotting nematodes^44^, soil nutrition^45^ and different subsoiler effects and compacted soil layers^46^. One can test whether the CRC shape would be horizontal in compacted soil and more vertical when the compacted layer has been broken. Additionally, the 3D CRC data would benefit documentation in large databases such as CassavaBase (https://www.cassavabase.org). However, it is important to note that our platform was developed for 3D modelling of the storage root, not for other root types of cassava, and detailed optimizations for both image acquisition of the root system and data extractions are required for such application.

## Conclusion

We provided an alternative approach to perform detailed CRC phenotyping in the field using 3D modeling. Our 3D platform is low cost, easy to set up, quick to operate in the field, yet provides high-quality 3D data with versatile plugin tools for 3D manipulations. Agisoft was selected for 3D reconstruction, and Blender was used for 3D analysis and data extraction. There are no requirements for sample transportation or a direct power line for the on-field operation, suitable with cassava field trials in remote cultivation areas. The platform has been demonstrated for assessing phenotypic variations of CRCs in a cassava population and, thus, can be used for genetic analysis and breeding for cassava.

## Supplementary Information

Supplementary Information 1.

Supplementary Information 2.

Supplementary Video 1.

Supplementary Video 2.

Supplementary Video 3.

Supplementary Video 4.

Supplementary Video 5.

## Data Availability

The datasets used and/or analysed during the current study are included in this published article and its Supplementary Information files. The dataset involving CRC phenotyping are available from the corresponding author on reasonable request.
