# Supplementary material for: Cassava root crown phenotyping using three-dimension (3D) multi-view stereo reconstruction
Source: Sci Rep. 2022 Jun 15;12:10030. doi: 10.1038/s41598-022-14325-4 (PMC9200979; doi:10.1038/s41598-022-14325-4)
Supplement: Supplementary file 1 — Supplementary Information 1. [file 41598_2022_14325_MOESM1_ESM.docx]

**Supporting Information**

Supplementary file S1. A 3D CRC file sample (.obj and .wmv formats)

Supplementary file S2. A video for 3D reconstruction of CRC using Agisoft Metashape Standard 1.6.5 (http://www.agisoft.com/downloads/installer/).

Supplementary file S3. A video for observing 3D CRCs in Blender analysis (Blender 2.90.1: http://www.blender.org).

Supplementary file S4. A video for the process of 3D CRC analysis using Blender (Blender 2.90.1: http://www.blender.org).

Supplementary File S5. VR for observing 3D CRCs.

Supplementary Table S1. Environment conditions at the cultivating site: Photharam District, Ratchaburi, Thailand (13.653699:99.821265). Data were obtained from a weather station closed to the plantation (Hydro-Informatics Institute, Thailand: https://govshare.data.go.th/explore).

| Period | Max-Min  temperature (°C) | Monthly rainfall (mm) |
| --- | --- | --- |
| Apr 19 | 45-25.8 | 0.8 |
| May 19 | 45-24.8 | 103.8 |
| Jun 19 | 45-25.8 | 79.4 |
| Jul 19 | 43-26.2 | 20 |
| Aug 19 | 41.5-25.8 | 67 |
| Sep 19 | 39-23.9 | 181 |
| Oct 19 | 39.6-23.2 | 194.6 |
| Nov 19 | 40.2-20 | 39.2 |
| Dec 19 | 39.5-15.5 | 0 |
| Jan 20 | 39.8-18.9 | 0 |
| Feb 20 | 39.8-18.4 | 0 |
| Mar 20 | 45-22 | 2.4 |
| Apr 20 | 43.5-23.1 | 60 |

Supplementary Table S2. Phenotypic data of 3D root crown training samples. The water volume (vol) was obtained from water replacement, and the 3D crown volume (3D vol) was calculated in 3D Blender. Delta (Δ) volume (vol) indicates percent differences between water replacement and 3D volumes. 3D area: 3D crown surface area, SV ratio: surface-to-volume ratio, DMC: dry matter content, starch FW: starch fresh weight, Ave-root length: average root length, Cy-soil vol: Cylinder soil volume, CRC comp: CRC compactness.

| ID line | Water vol  (cm^3^) | 3D  vol (cm^3^) | Δ vol (%) | 3D area (cm^2^) | Root weight (kg) | SV  ratio | Root density | DMC (%) | Mois  ture (%) | Starch FW (%) | Root  # | Root angle  (°) | Crown diameter (cm) | Ave-root length (cm) | Cy-soil vol  (x1000 cm^3^) | CRC comp (%) |
| --- | --- | --- | --- | --- | --- | --- | --- | --- | --- | --- | --- | --- | --- | --- | --- | --- |
| 813-1 | 4,242 | 4,537 | 7 | 3,986 | 4.50 | 0.88 | 0.99 | 38.67 | 61.33 | 21.68 | 13 | 94 | 81.94 | 28.04 | 147.18 | 3.08 |
| 813-2 | 3,470 | 3,681 | 6 | 3,509 | 3.70 | 0.95 | 1.01 | 37.00 | 63.00 | 22.31 | 12 | 138 | 66.99 | 24.73 | 95.58 | 3.85 |
| 813-3 | 3,450 | 3,502 | 2 | 4,016 | 3.50 | 1.15 | 1.00 | 32.33 | 67.67 | 19.56 | 13 | 101 | 64.13 | 26.86 | 89.31 | 3.92 |
| KU50-1 | 2,320 | 2,348 | 1 | 2,318 | 2.30 | 0.99 | 0.98 | 37.00 | 63.00 | 23.97 | 9 | 85 | 63 | 31.57 | 126.29 | 1.86 |
| KU50-2 | 2,620 | 2,644 | 1 | 2,623 | 2.70 | 0.99 | 1.02 | 38.67 | 61.33 | 24.35 | 9 | 127 | 58.88 | 24.10 | 59.69 | 4.43 |
| KU50-3 | 2,475 | 2,503 | 1 | 2,626 | 2.60 | 1.05 | 1.04 | 37.33 | 62.67 | 24.03 | 12 | 106 | 58.63 | 22.87 | 65.84 | 3.80 |
| 86-1 | 5,240 | 5,361 | 2 | 5,258 | 6.10 | 0.98 | 1.14 | 37.67 | 62.33 | 22.82 | 14 | 100 | 81.41 | 32.12 | 195.81 | 2.74 |
| 86-2 | 3,590 | 3,350 | -7 | 3,209 | 4.10 | 0.96 | 1.22 | 37.00 | 63.00 | 24.77 | 10 | 115 | 66.83 | 28.89 | 170.43 | 1.97 |
| 86-3 | 2,190 | 2,218 | 1 | 2,585 | 2.10 | 1.17 | 0.95 | 32.33 | 67.67 | 20.73 | 11 | 68 | 67.92 | 27.80 | 80.39 | 2.76 |
| 899-1 | 4,550 | 4,625 | 2 | 4,220 | 5.30 | 0.91 | 1.15 | 41.00 | 59.00 | 26.81 | 11 | 146 | 72.41 | 28.77 | 152.71 | 3.03 |
| 899-2 | 4,090 | 4,044 | -1 | 4,488 | 4.50 | 1.11 | 1.11 | 35.33 | 64.67 | 22.39 | 14 | 107 | 91.18 | 29.13 | 201.04 | 2.01 |
| 899-3 | 3,900 | 4,066 | 4 | 3,762 | 4.60 | 0.93 | 1.13 | 39.33 | 60.67 | 25.62 | 14 | 120 | 80.89 | 33.82 | 132.14 | 3.08 |
| 88-1 | 4,400 | 4,387 | 0 | 3,729 | 4.90 | 0.85 | 1.12 | 36.33 | 63.67 | 24.59 | 18 | 113 | 55.21 | 21.58 | 71.47 | 6.14 |
| 88-2 | 5,240 | 5,711 | 9 | 5,500 | 5.90 | 0.96 | 1.03 | 38.67 | 61.33 | 26.63 | 17 | 123 | 64.37 | 29.34 | 127.53 | 4.48 |
| 88-3 | 4,090 | 3,709 | -9 | 3,792 | 4.60 | 1.02 | 1.24 | 34.67 | 65.33 | 22.06 | 17 | 91 | 62.84 | 24.62 | 89.97 | 4.12 |
| 824-1 | 6,525 | 6,679 | 2 | 6,688 | 7.10 | 1.00 | 1.06 | 31.67 | 68.33 | 18.09 | 17 | 121 | 111 | 40.03 | 176.43 | 3.79 |
| 824-2 | 5,640 | 6,169 | 9 | 5,622 | 6.10 | 0.91 | 0.99 | 32.67 | 67.33 | 20.04 | 18 | 121 | 77.85 | 34.23 | 226.16 | 2.73 |
| 83-1 | 3,540 | 3,589 | 1 | 4,309 | 3.70 | 1.20 | 1.03 | 39.00 | 61.00 | 27.38 | 12 | 110 | 87.8 | 32.57 | 212.18 | 1.69 |
| 83-3 | 2,040 | 2,206 | 8 | 2,885 | 2.10 | 1.31 | 0.95 | 34.00 | 66.00 | 20.08 | 11 | 116 | 66.12 | 29.78 | 117.73 | 1.87 |

Supplementary Table S3. Analysis of variance for nine 3D traits, root weight and HI in S_1_ and S_2_ populations. Mean squares of treatment adjusted are presented.

| Source | df | 3D crown vol  (x1,000 cm^3^) | 3D crown area  (x1,000 cm^2^) | SV  ratio | Crown diameter (cm) | Root angle (°) | Root weight (kg) | HI | Root density | Root number | Cylinder soil volume (x1000 cm^3^) | CRC compactness (%) |
| --- | --- | --- | --- | --- | --- | --- | --- | --- | --- | --- | --- | --- |
| Block (ignoring Treatments) | 8 | 7.42^***^ | 2.348^***^ | 0.03004^***^ | 152.4 ^NS^ | 98 ^NS^ | 7.43^*^ | 0.01389 ^NS^ | 0.03249 ^NS^ | 5.181^*^ | 3.002e+09 ^NS^ | 4.105 ^NS^ |
| Treatment (eliminating Blocks) | 45 | 9.73^***^ | 2.518^***^ | 0.03283^***^ | 247 ^NS^ | 103 ^NS^ | 8.74^**^ | 0.02713 ^NS^ | 0.08319^**^ | 4.217^*^ | 3.677e+09^*^ | 4.668 ^NS^ |
| Treatment: Check | 4 | 55.95^***^ | 10.409^***^ | 0.11641^***^ | 154.6 ^NS^ | 439.6^**^ | 32.28^***^ | 0.09312^**^ | 0.10337^*^ | 8.012^**^ | 2.596e+10^***^ | 12.291^*^ |
| Treatment: Test and Test vs. Check | 41 | 5.22^***^ | 1.748^***^ | 1.0116^***^ | 256 ^NS^ | 70.2 ^NS^ | 6.45^*^ | 0.20819 ^NS^ | 0.08122^**^ | 3.847^*^ | 2.047e+09 ^NS^ | 4.111 ^NS^ |
| Residuals | 33 | 1.2 | 0.471 | 0.074 | 257 | 106.5 | 3.24 | 0.01569 | 0.03365 | 1.984 | 1.648e+09 | 3.706 |

df: degrees of freedom, NS: non-significant. *, **, *** represent significant at P< 0.05, 0.01 and 0.001, respectively.

Supplementary Table S4. Analysis of variance for nine 3D traits, root weight and HI in S_1_ and S_2_ populations. Mean squares of block adjusted are presented.

| Source | df | 3D crown vol  (x1,000 cm^3^) | 3D crown area  (x1,000 cm^2^) | SV  ratio | Crown  diameter (cm) | Root angle (°) | Root weight (kg) | HI | Root density | Root number | Cylinder soil volume (x1000 cm^3^) | CRC compactness (%) |
| --- | --- | --- | --- | --- | --- | --- | --- | --- | --- | --- | --- | --- |
| Treatment (ignoring Blocks) | 45 | 10.74^***^ | 2.87^***^ | 0.0369^***^ | 226.1^NS^ | 106.7 ^NS^ | 9.06^**^ | 0.028^*^ | 0.08426^**^ | 4.695^**^ | 3.998e+09^*^ | 4.8 ^NS^ |
| Treatment: Check | 4 | 55.95^***^ | 10.41^***^ | 0.1164^***^ | 154.6 ^NS^ | 439.6^**^ | 32.28^***^ | 0.09312^**^ | 0.10337^*^ | 8.012^**^ | 2.596e+10^***^ | 12.29^*^ |
| Treatment: Test | 40 | 2.75^**^ | 1.13^**^ | 0.0187^***^ | 198.1 ^NS^ | 73.5 ^NS^ | 3.73 ^NS^ | 0.02025 ^NS^ | 0.08415^**^ | 3.785^*^ | 2.016e+09 ^NS^ | 3.36 ^NS^ |
| Treatment: Test vs. Check | 1 | 149.59^***^ | 42.31^***^ | 0.4482^***^ | 1630.2^*^ | 103 ^NS^ | 129.6^***^ | 0.07760^*^ | 0.01214 ^NS^ | 27.828^***^ | 1.742e+10^**^ | 40.20^**^ |
| Block | 8 | 1.72^NS^ | 0.37 ^NS^ | 0.0069^*^ | 270.2 ^NS^ | 77.1 ^NS^ | 5.62 ^NS^ | 0.00897^NS^ | 0.02647 ^NS^ | 2.492 ^NS^ | 1.238e+09 ^NS^ | 3.36 ^NS^ |
| Residuals | 33 | 1.2 | 0.47 | 0.0022 | 257 | 106.5 | 3.24 | 0.01569 | 0.03365 | 1.984 | 1.648e+09 | 3.71 |

df: degrees of freedom, NS: non-significant. *, **, *** represent significant at P< 0.05, 0.01 and 0.001, respectively.

Supplementary Figure S1. CRC images for photogrammetry and photo shooting setup. Representative CRC images taken from four angles are presented. The root crown object was placed on a green background (120 x 120 cm) with a reference object using a card box.

Supplementary Figure S2. Evaluations of five photogrammetry software for 3D reconstruction. Six parameters were tested for selecting the most suitable software for reconstructions of CRCs. Testing was performed using 40 images per root crown, and at least 21 root crown datasets were performed.

Supplementary Figure S3. Evaluations of five 3D modelling software for CRC analysis. Four parameters were used for selecting the most suitable software for extracting phenotypic traits from 3D crown models.

Supplementary Figure S4. (a) Separated parts of storage roots and a stem section from a CRC in Blender (Blender 2.90.1: http://www.blender.org). (b-c) An individual root and root length analysis. (d) Analysis of a root diameter.
